# Scoping Review on True and Relative Bradycardia in Trauma: How to Approach Bradycardia in Traumatic Brain Injury

**DOI:** 10.1007/s12265-026-10772-w

**Published:** 2026-04-27

**Authors:** Ayman El-Menyar, Naushad A. Khan, Abdul Rehman Abid, Eman Elmenyar, Hassan Al-Thani

**Affiliations:** 1 Trauma and Vascular Surgery, Clinical Research, Hamad Medical Corporation (HMC), PO Box 3050, Doha, Qatar; 2 Department of Clinical Medicine, Weill Cornell Medical School, PO Box 24144, Doha, Qatar; 3 Cardiology Department, Heart Hospital, Hamad Medical Corporation (HMC), PO Box 3050, Doha, Qatar; 4 Faculty of Medicine, Bahçeşehir University, Istanbul, Turkey; 5 Department of Surgery, Vascular Surgery, HMC, PO Box 3050, Doha, Qatar

**Keywords:** Traumatic brain injury, Cushing reflex, Relative bradycardia, True bradycardia, Intracranial pressure, Neuro-cardiology

## Abstract

Brain–heart interactions have garnered considerable interest in the landscape of traumatic brain injury (TBI). This scoping review focused on bradycardia caused by high intracranial pressure (ICP), brainstem injury, autonomic imbalance, hypothalamic–pituitary–adrenal axis disruption, or massive bleeding. The latter condition is attributed to a physiological phenomenon called relative bradycardia (RB). True bradycardia results from underlying physiological or pathological cardiac disorders, whereas RB has different definitions and implications in medical and surgical settings. The former reflects pulse-temperature dissociation, while the latter reflects pulse-pressure dissociation. However, it can reflect an abnormal neurological response called the Cushing reflex. Therefore, bradycardia may indicate imminent shock after TBI associated with torso injuries or high ICP following severe isolated TBI. RB is underrecognized and underappreciated in emergency settings. This review investigated whether RB affects patient survival and neurological function. Physicians should approach patients presenting with RB with a high index of suspicion and timely management.

**Graphical Abstract:**

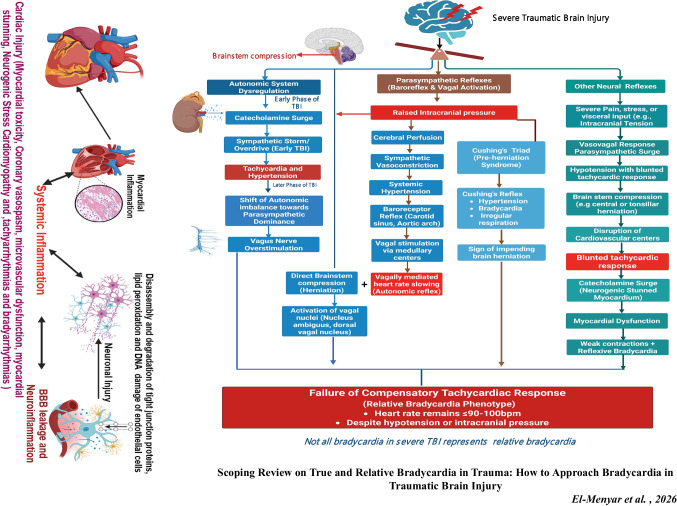

## Introduction

Traumatic brain injury (TBI) is a significant and pressing global public health concern. Moreover, TBI remains the leading cause of death and disability, accounting for 30% of all injury-related deaths [1]. The complex interaction between the brain and heart has gained increasing recognition in the scientific community. This brain–heart interaction (BHI) involves complex neuro-cardiac dynamics and pathophysiological connections between the central nervous system (CNS) and the cardiovascular system (CVS) [2, 3]. This dynamic cross-talk can result directly from stimulation of specific brain regions either sympathetic activation (the fight-or-flight response) or parasympathetic activation (the rest-and-digest response) [4, 5].

Furthermore, the neuroendocrine responses are also involved in this bidirectional relationship, which can result in clinical syndromes such as "sympathetic storm" [6, 7]. This syndrome can lead to severe abnormalities in heart rate (HR), hemodynamic instability, impaired cerebral perfusion pressure (CPP), and cerebral hypoperfusion [8]. Maintaining appropriate CPP is critical in managing patients with TBI. CPP is calculated as the difference between the mean arterial pressure (MAP) and the intracranial pressure (ICP). The MAP is influenced by HR, stroke volume (SV), and systemic vascular resistance (SVR), and is calculated as the cardiac output (CO) multiplied by the SVR. As the CO is a product of HR and SV, abnormalities in HR can negatively affect the CPP. Too slow or too fast HR may reduce the SV and systemic blood pressure, leading to symptomatic hypotension and organ malperfusion.

**Fig. 1:**
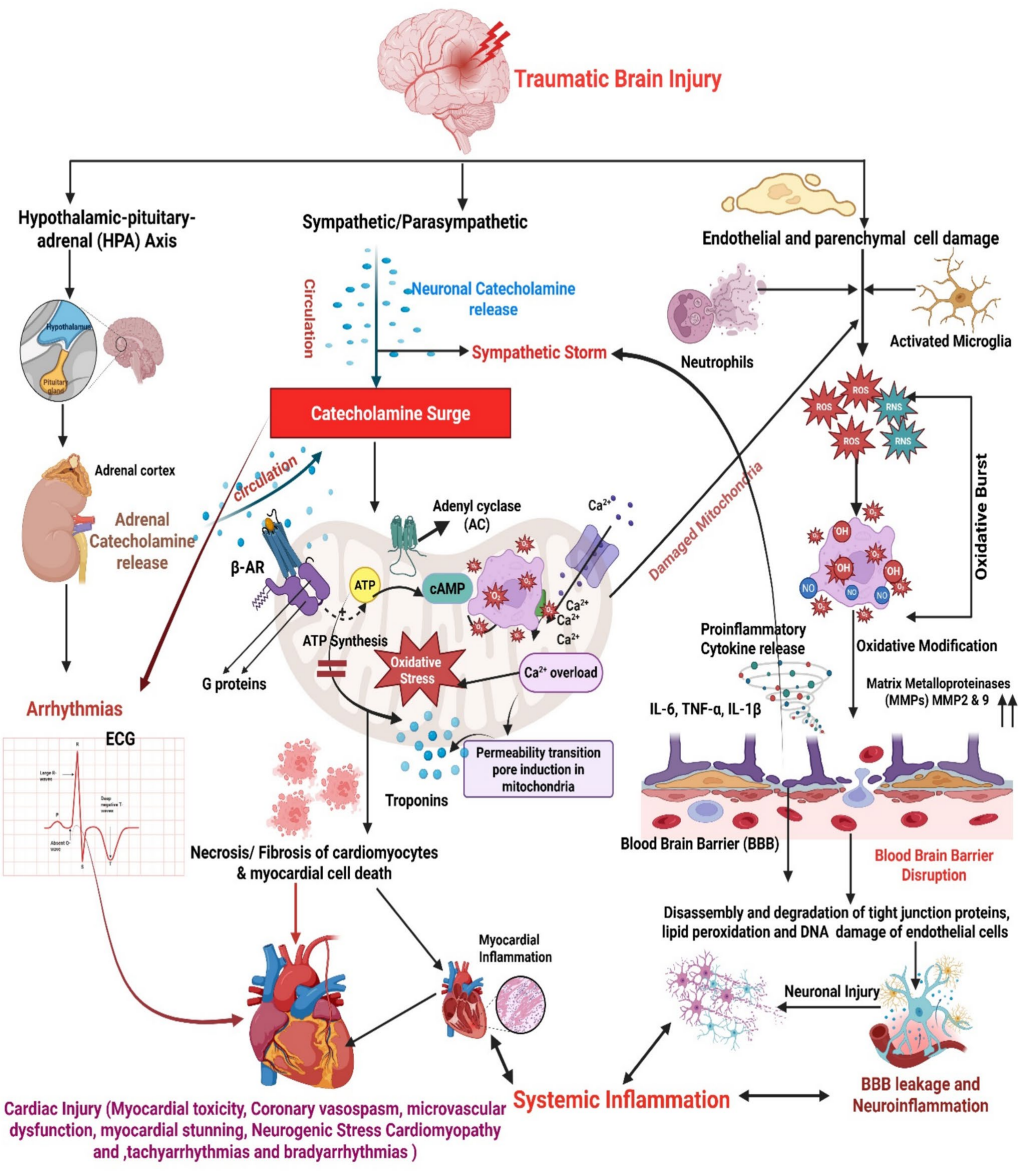
Traumatic brain injury (TBI) sets off a chain reaction of systemic processes outside the central nervous system that severely compromise heart function. The primary mechanical damage disrupts the blood–brain barrier (BBB), thereby allowing inflammatory mediators and peripheral immune cells to infiltrate the brain parenchyma. Reactive oxygen species (ROS) production initiates an intense oxidative burst, leading to mitochondrial damage, oxidative stress, and progressive neuronal damage. Parallel to oxidative damage, TBI stimulates the sympathetic nervous system, resulting in a significant surge of catecholamines via both adrenal and neural pathways. Although initially protective, excessive catecholamine levels overexcite beta-adrenergic receptors in the heart, therefore generating calcium overload, mitochondrial damage, and direct myocardial toxicity. At the same time, systemic inflammatory cytokines, including IL-1β, IL-6, and TNF-α, exacerbate myocardial stress, thereby promoting inflammation and dysfunction. Neurogenic stress cardiomyopathy, defined by myocardial stunning, lower ejection fraction, and arrhythmias, results from the confluence of catecholamine toxicity and systemic inflammation. Notably, the stressed myocardium typically exhibits reversible damage if the acute period is endured. As cardiac dysfunction worsens, Arrhythmias, including tachyarrhythmias and bradyarrhythmia, become more prevalent. Notably, brainstem autonomic dysregulation, vagal overactivation (Cushing reflex), or cardiac conduction anomalies related to heart damage might cause bradycardia. Figure created in https://BioRender.com

Furthermore, in severe cases, systemic and cerebral inflammation, as well as disruption of the blood–brain barrier (BBB), which occurs during TBI, may lead to neurogenic stress cardiomyopathy, myocardial stunning, destabilizing systemic hemodynamics, and worsening cerebral perfusion [9, 10]. Following TBI, secondary cerebral injuries like intracranial hypertension, seizures, and tissue hypoxia are well known; however, extracranial complications, especially cardiac dysfunction, play a crucial role in worsening the outcome [11]. The pathophysiology of cardiac injury within the spectrum of TBI is complex (Fig. 1**)** and includes autonomic dysregulation, catecholamine surges, and systemic inflammatory responses, which can lead to HR abnormalities, a typical phenomenon in TBI patients and often associated with sympathetic nervous system hyperactivity [12].

Several factors can explain the inadequate chronotropic response (bradycardia). Examples include dominance of vagal response, neurogenic shock, hypothermia, beta blocker use, atherosclerosis, conduction defects, Cushing reflex, spinal cord injury, cardiac tamponade, and imminent shock status [13, 14]. True bradycardia (TB) is a form of bradycardia characterized by a HR ≤ 60 bpm and results from direct suppression of the sinoatrial node (SAN) in the heart. In this condition, systolic blood pressure (SBP) begins to decrease in the same direction as the slow HR after failure of the compensatory mechanisms. This can occur due to physiological or pathological cardiac disorders, as well as the effects of certain drugs or toxins. In these cases, the SBP responds in parallel to the significant decrease in HR. The second form of bradycardia is called relative bradycardia (RB), which reflects the inability of HR to respond to changes in SBP or body temperature. In non-trauma medical situations, RB refers to pulse-temperature dissociation. In trauma settings, it reflects pulse-SBP dissociation (failure to attain a tachycardic response after trauma-induced hypotension) [15].

As shown in Table 1, in RB, the HR can be ≤ 90 or ≤ 100 bpm, and the simultaneous SBP can be ≤ 90 or ≤ 100 mmHg, respectively [13, 15–19]. In most people, once SBP decreases (primary or independent variable) for a reason such as bleeding, the HR (secondary or dependent variable) will start increasing (compensatory process) to maintain the SV and organ' perfusion within a limit. Therefore, bradycardia is not a usual response in most subjects sustaining bleeding following trauma. However, in almost one-third of subjects, the tachycardia response to bleeding is absent, reflecting the dissociation between HR and SBP. Moreover, TBI can present in isolation or as part of polytrauma with internal bleeding; therefore, identifying the cause of bradycardia (TB or RB) is clinically significant, as the implications would differ and be time-sensitive. In isolated TBI, bradycardia could reflect pathological rather than physiological responses. Rarely, authors used the term (RB) when HR is < 90 bpm in patients with significant bleeding, while others used the term “paradoxical bradycardia” if the HR < 60 bpm, and some used the term “absence of tachycardic response” [13, 20–23].

**Table 1:** True and relative bradycardia in overall trauma and traumatic brain injury contexts

Author	Study Design	True (TB) or Relative Bradycardia (RB)	Population	Comments
Sijercic et al. 2018 [35]	Prospective	TB	104 SAH patients (spontaneous SAH and SAH following TBI)	Sinus bradycardia was predominant in the TBI group (*p* = 0.05)
Ley et al. 2010 [65]	Retrospective	TB	11,977 patients with moderate to severe isolated TBI	HR < 50,50–59,60–69, and > 110 were independent predictors of increased mortality HR < 50 bpm: AOR 4.70 (95% CI 3.24–6.83, *p* < 0.0001) HR 50–59 bpm: AOR 2.21 (95% CI 1.60–3.07, *p* < 0.0001)
Ley et al. 2009 [17]	Retrospective	RB^1^ vs Tachycardia^1^	130,906 Adult trauma patients	Incidence of RB was 1.2% in all traumas, and 44% in hypotensive trauma patients. Mortality was significantly higher in the RB group (30.1% vs. 22.6%) except for older patients (age ≥ 55) and patients with a Glasgow coma scale score ≥ 12
Lenstra et al. 2021 [83]	Retrospective	TB	198/298 severe TBI patients with available ECG recordings	Sinus bradycardia in 31% Patients with arrhythmia had a higher ISS (*p* = 0.04) An increase in grading of diffuse brain injury was associated with more arrhythmias (*p* = 0.04)
Poudel et al. 2023 [77]	Retrospective cross-sectional study	TB	45 patients with SAH	24% with sinus bradycardia Isolated rhythm abnormality was observed in 6 patients, all presenting as bradycardia
Subramanium et al. 2015 [15]	Retrospective	TB and RB^2^	437 patients with severe TBI	RB in 48% of hypertensive group 9% presented with TB and showed a higher mortality rate. Mortality: 71% in HR ≤ 60 vs. 27% in HR > 60; *p* < 0.001RB: unable to identify HR between 60 and 90 as a predictor of mortality (*p* = 0.113), but HR ≤ 60 was significant (*p* = 0.006)
Gibbons et al. 2020 [82]	Retrospective analysis of prospectively collected data	TB	326 patients with moderate to severe TBI	11% had symptomatic bradycardia
Shaikh et al. 2016 [84]	Prospective	TB	138 patients with high spinal cord injury	33% had prolonged bradycardia Higher asystole, complications, ISS score (*P* = 0.02), ICU (*P* = 0.001) and hospital stay (*P* = 0.002)
Baffoun et al. 2011[85]	Prospective	TB	35 patients with traumatic SAH	4 patients had bradycardia
Hamila et al. 2022 [86]	Prospective	TB	50 patients with TBI	Sinus bradycardia occurred in 18% on admission and 5% at 24 and 72 h after admission. A significant association was found between ECG changes and brain edema, intracerebral hemorrhage, and subarachnoid hemorrhage
Yumoto et al. 2018 [19]	Retrospective	TB and RB^3^	297 patients requiring neurological intervention (2 and 15 years old)	Bradycardia in ages 7–10 and 11–15 was associated with brain injury requiring immediate neurosurgical intervention (BI-NSI) RB revealed that higher SBP and lower HR were significant predictors for BI-NSI in a group of 7–10 years of age and 11–15 years of age
Barriot and Riou 1987 [13]	Retrospective	RB^4^ & PB	273 acute hemorrhagic shock: 20 had PB and 0 had RB	7% had PB and none had RB. HR in PB is a reliable indicator of hypovolemia correction, and atropine may be inappropriate as it can mask inadequate fluid loading
Demetriades et al. 1998 [16]	Retrospective	RB^5^	10,833 major trauma patients	2% had RB in overall and 29% in hypotensive trauma patients. Overall, the crude mortality was 29.2% among tachycardia patients and 21.7% among bradycardia patients
Thompson et al. 1990 [18]	Retrospective	RB^6^	256 patients with isolated penetrating abdominal trauma and 938 patients with isolated severe extremity trauma	Incidence of RB (1.8% to 3.1%) depends on the type of trauma, and 35% of all hypotensive trauma patients

RB^1^: SBP ≤ 90 mmHg with HR ≤ 90 vs tachycardia (SBP ≤ 90 with HR > 90 bpm)

RB^2^: HR ≤ 90 bpm among patients with SBP ≥ 140

RB^3^: higher SBP and lower HR in children with TBI

RB^4^: paradoxical bradycardia (PB):(SBP ≤ 70 and HR ≤ 60, relative bradycardia (RB):(SBP ≤ 70 and HR 60 to 100 bpm)

RB^5^: SBP ≤ 90 mm Hg and a HR ≤ 90

RB^6^: HR < 100 with a concomitant SBP < 100 mm Hg

This scoping, contemporary review synthesizes current evidence on the underlying pathophysiology and clinical relevance of BHI following TBI, with particular emphasis on bradycardia and its two phenotypes TB and RB), especially RB in the context of TBI. The literature search was conducted in English-language sources using PubMed, Google Scholar, and Scopus, covering publications from database inception through June 2025. The association between bradycardia and outcomes will be assessed to establish evidence of whether RB is associated with a higher mortality rate and to investigate potential links between HR and secondary outcomes, such as neurological function and ICU length of stay in the trauma settings.

## Discussion

RB is underrecognized in medical and surgical settings, mainly because it contradicts the expected compensatory tachycardic response to hemorrhage and may therefore be misinterpreted as physiological stability during early triage and resuscitation. Several trauma studies have demonstrated that a substantial proportion of hypotensive patients present with RB or an absent tachycardic response, a phenomenon described as “paradoxical bradycardia,” yet it remains variably defined and inconsistently emphasized in clinical algorithms and guidelines [13, 16–18, 24].

The underlying mechanism of RB is not well understood; however, in medical conditions, it can be attributed to the release of inflammatory cytokines, increased vagal tone, a direct pathogenic effect on the myocardium, and electrolyte abnormalities [24]. It is a sensitive bedside clinical sign in medical conditions. In septic shock, it could be an indicator of lower mortality [25].

### Bradycardia in Polytrauma With Borderline Blood Pressure or Hypotension

The leading cause of preventable death in polytrauma patients is bleeding, accounting for 30–40% of trauma deaths [26]. It should be detected and managed promptly, as it is a major contributor to early mortality. In blunt trauma, tachycardia and hypotension are alarming for internal bleeding, however some patients presenting with imminent shock may have borderline blood pressure with the absence of tachycardia response [27]. Therefore, RB helps flag high-risk patients and calls for better triage and aggressive treatment. The incidence of RB among overall trauma patients varied in the literature as 1.2%, 2%, or 3% [16–18], while in hypotensive trauma patients, it was reported as 7%, 29%, 35% or 44% [13, 16–18]. RB is common in adult trauma patients presenting with hypotension and is associated with higher mortality [17, 24].

However, in the prior six studies reporting on RB, there was inconsistency in definitions and cutoffs, as shown in Table 1. Two studies used “90” as the cutoff for HR and SBP, while one study used “100” for both parameters [9, 18, 24]. Using a lower cut-off will ensure that more sicker patients are included, at the expense of lower specificity and fewer false-negative cases, and higher sensitivity and more false-positive cases. While choosing a higher cutoff decreases sensitivity and false-positive cases, it increases specificity and false-negative cases. If the outcome includes mortality, it is better to have fewer false-negative cases (i.e., a lower cutoff) [28, 29]. On the other hand, if the outcome is a lifesaving intervention, a higher cutoff may be better, but it may include unnecessary activation of massive transfusion protocol (MTP).

The initial treatment of RB in acute hemorrhage is not the treatment of bradycardia itself, but rather rapid and massive fluid/blood resuscitation, as atropine use may be misleading and detrimental at this stage [13]. Whereas in isolated and/or severe TBI, the primary treatment is to reduce the high ICP medically or through decompressive craniotomy.

RB can be partly explained by the fact that one-third of people have a low tolerance to blood volume reduction due to greater vagal withdrawal of baroreflex sensitivity (i.e., a low baseline baroreflex sensitivity) after bleeding [17]. In these individuals, the afferent branches of the glossopharyngeal and vagal nerves in the aortic arch responded to a progressive decrease in pulse pressure during the early phase of bleeding. Upon activation of these baroreceptors, the efferent vagal activity is blunted, causing tachycardia. However, once the subject loses one-fifth or more of their blood volume, the efferent branches of the vagus nerve signal increase, leading to a slow HR (i.e., RB) and increased ventricular diastolic filling time [14].

Several hypotheses have been proposed regarding the fundamental mechanisms of RB. According to Barriot et al., the body may over-activate the parasympathetic system in response to rapid and severe bleeding, thereby preventing the expected tachycardic response [13]. Snyder and Dresnik suggested that intraperitoneal hemorrhage triggers parasympathetic reflexes, resulting in bradycardia [23]. Oberg et al. characterized RB as a vagally mediated reflex derived from left ventricular mechanoreceptors. Bradycardia in animal models was shown to follow an initial tachycardic reaction after bleeding, presumably to increase diastolic filling and preserve stroke volume. The reflex disappeared after the severance of the vagus nerve, which confirmed its parasympathetic origin [30].

**Fig. 2:**
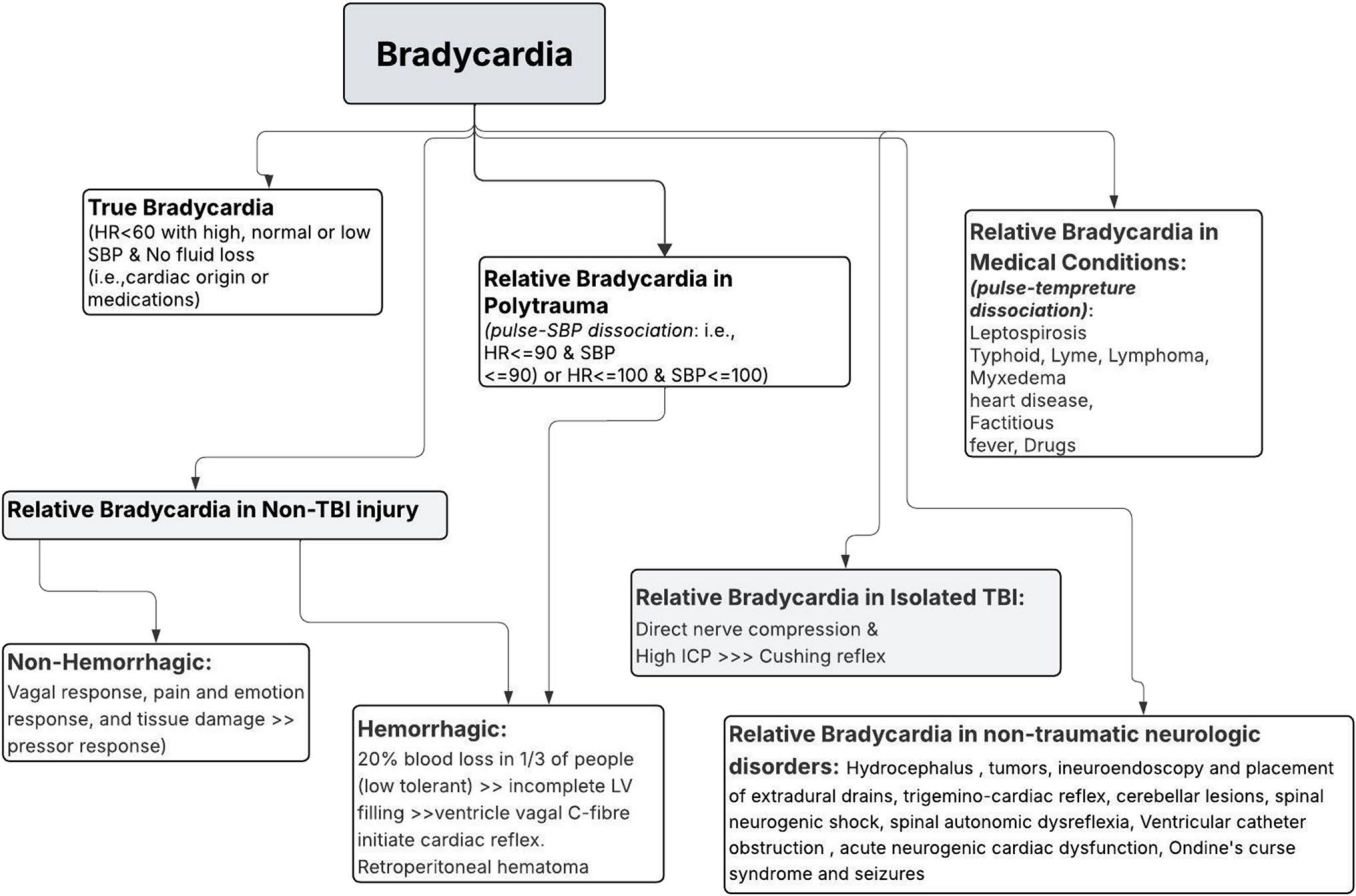
**Definition and possible underlying mechanisms of true and relative bradycardia**

**Fig. 3:**
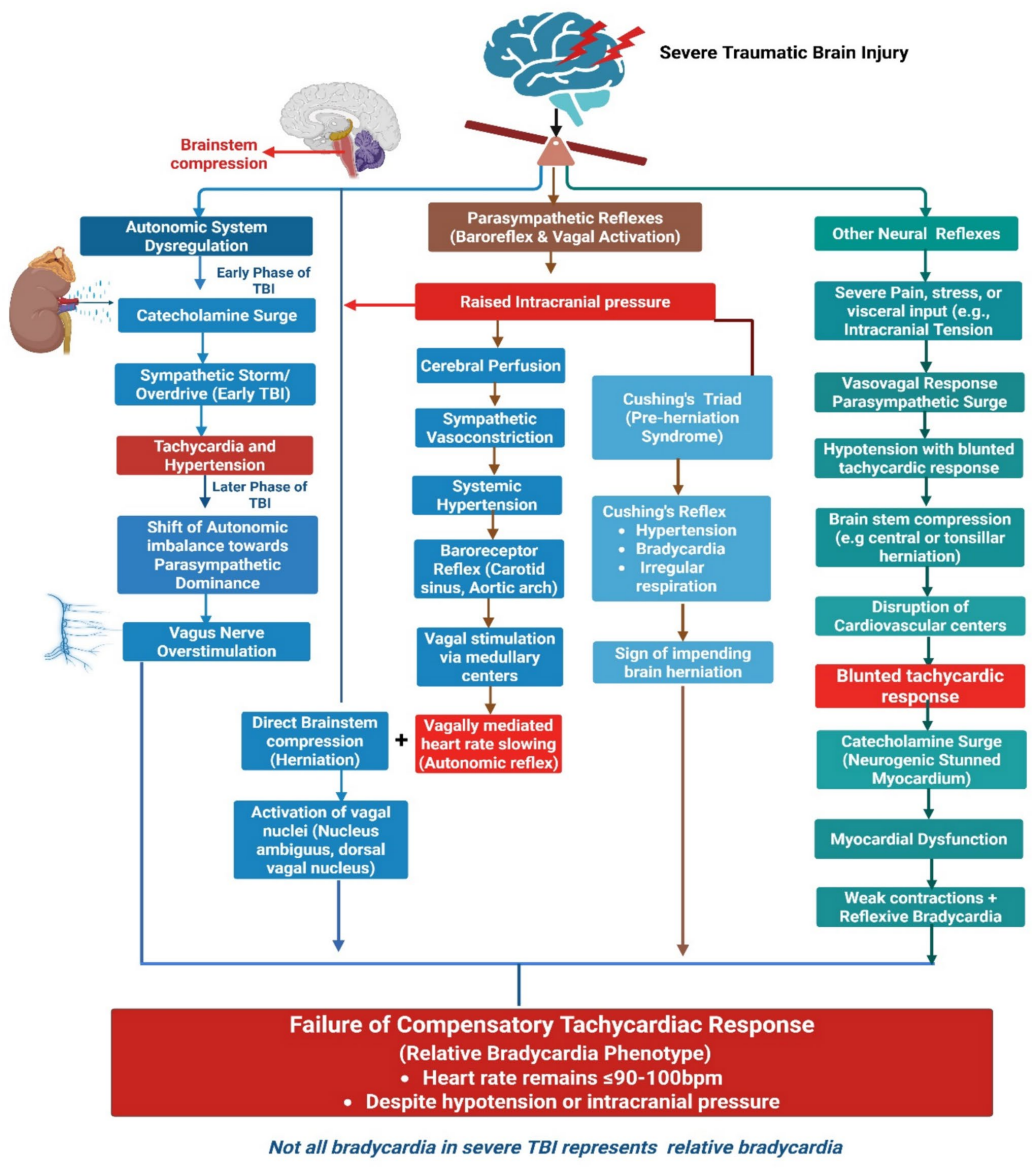
**Autonomic and Neurogenic Mechanisms Contributing to an Inadequate Heart Rate Response in Severe TBI**: TBI leads to increased intracranial pressure and brainstem compression, resulting in autonomic imbalance. Sympathetic activation may produce systemic hypertension, triggering baroreceptor-mediated vagal over-activity and an inadequate compensatory heart rate response. In this context, the relative bradycardia (RB) phenotype reflects neurogenic and autonomic dysfunction rather than primary cardiac pathology and may serve as an early indicator of worsening intracranial dynamics. Importantly, this framework describes failure of the expected tachycardic response and does not imply that all bradycardia observed in TBI represents RB, as heart rate changes may manifest as true or RB depending on clinical context. F

Figure 2 shows the definitions and underlying mechanisms of TB and RB. Usually, acute trauma triggers sympathetic activation, resulting in an elevated HR as a part of the stress response. However, in patients with severe TBI, RB often occurs, particularly when accompanied by signs of increased ICP [16, 31]. It is commonly observed as part of Cushing’s triad, a classic warning of potential brain herniation. Unlike reflex tachycardia from hypovolemia or pain, RB in TBI suggests autonomic dysregulation or brainstem involvement, underscoring its clinical significance [19]. Other proposed mechanisms include non-hemorrhagic vagal reflexes and reduced baroreflex buffering capacity. These autonomic disturbances reflect critical intracranial pathology and often precede neurological deterioration. Figure 3 illustrates the autonomic and neurogenic mechanisms that may contribute to an RB phenotype in severe TBI.

### Bradycardia in Isolated TBI

During TBI management, physicians primarily use clinical markers to detect a sudden rise in ICP. Low Glasgow Coma Scale (GCS), fixed and dilated pupils, and Cushing's triad are indicators of elevated ICP. These indicators are often used to determine whether to initiate temporary ICP-lowering treatments [32]. Bradycardia involves overactivation of the parasympathetic nervous system [33]. This condition is particularly pertinent in TBI due to its association with increased ICP and brainstem dysfunction, which complicate clinical management [33]. The physiological underpinnings of bradycardia in TBI may exacerbate cerebral hypoperfusion as a result of secondary hypotension and worsen secondary ischemic brain damage, thereby complicating the clinical course of TBI and posing significant challenges for timely diagnosis and effective management [34].

Studies investigating bradycardia in TBI are scarce. However, the available research suggests a significant correlation between bradycardia (TB or RB) and poor clinical outcomes. A study by Sijercic et al. reported that electrocardiographic (ECG) abnormalities such as atrial fibrillation and sinus bradycardia were common among TBI patients with a significant correlation with mortality [35]. Moreover, Kerbs et al. reported that initial bradycardia, along with low GCS and hypoxia, were predictors of mortality in TBI patients [36]. Similarly, Barriot et al. reported that RB was associated with increased mortality and considered a pre-terminal event [13]. However, further research is needed to elucidate the underlying mechanisms and determine whether bradycardia is a potential therapeutic target in TBI management.

### Autonomic Nervous Dysfunction and Bradycardia

The autonomic nervous system (ANS), comprising the sympathetic and parasympathetic systems, regulates various organ functions and is crucial for maintaining normal cardiac function. Autonomic dysfunction is a common post-traumatic brain injury outcome that affects cardiovascular, inflammatory, and metabolic systems [37]. This dysregulation is often associated with altered baroreflex sensitivity, a hallmark of TBI-related ANS dysregulation, along with impaired sympathetic and parasympathetic balance and blood flow autoregulation, compromising brainstem-mediated regulation of HR and vascular resistance, leading to autonomic instability and increased risk of early mortality [38]. One key manifestation of TBI-induced ANS dysfunction is bradycardia, attributed to increased vagal activity, particularly in patients with brainstem injury or elevated ICP [39]. This cardiac dysregulation worsens cerebral hypoperfusion due to the defect in cerebral blood flow autoregulation, thereby worsening the neurological status [40]. In addition, autonomic imbalance in TBI has been linked with neuroinflammation, oxidative stress, BBB disruption, and unfavorable outcome [41, 42]. Post-TBI sympathetic hyperactivity may also suppress immune functions and disrupt the hypothalamic–pituitary–adrenal (HPA) axis, thereby increasing the patient's susceptibility to secondary infections and metabolic complications [43].

### Neuro-Cardiac Impact of TBI: The Path to Bradycardia

There are several causes of bradycardia in patients with neurological insults such as space occupying lesions initiating Cushing reflex (CR) (hydrocephalus, subdural hematoma, tumors,), interventions such as neuroendoscopy and placement of extradural drains, trigemino-cardiac reflex (TCR), cerebellar lesions, spinal neurogenic shock, spinal autonomic dysreflexia, Ventricular catheter obstruction, acute neurogenic cardiac dysfunction, Ondine's curse syndrome and seizures [33].

#### Cushing’s Reflex (CR)

TBI significantly impacts cardiovascular regulation through disruption of neuro-cardiac reflexes, which link the CNS to autonomic heart control. This dysregulation can lead to bradycardia, tachycardia, or cardiac arrest. Among these reflexive responses, the CR, also known as Cushing's triad, is a significant sign in the context of TBI. This CR is the body’s protective response to increased ICP, aiming to preserve cerebral blood flow to prevent ischemia. It manifests as bradycardia, caused by heightened vagal tone; hypertension, driven by systemic vasoconstriction to sustain cerebral perfusion; and respiratory irregularities due to brainstem compression. However, as the brainstem becomes compressed, autonomic regulation and respiratory control are further disrupted, leading to irregular breathing patterns. This reflex is a critical indicator of severe TBI and conditions like intracranial hemorrhage (ICH).

CR is a critical indicator of increased ICP and may signal the development of brain herniation and mortality [44, 45]. A rapid increase in the ICP triggers CR, and warrants urgent clinical evaluation and timely intervention within a comprehensive neurocritical care pathway, which may include medical management and, in selected cases, decompressive craniectomy (DC). Jo et al. [46] suggested that a change in HR in response to a change in mean atrial pressure after DC may indicate preserved vasomotor reflexes and autoregulation. Their study demonstrated that while ICP decreased in all patients after craniectomy, the HR response correlated with the clinical outcomes. Interestingly, patients who had a decrease in HR (or an unchanged HR) post-DC were more likely to have a higher mortality risk than those who had an increase in HR.

#### Mechanisms Linking Elevated ICP and RB

The mechanism involves an increase in ICP, which compresses cerebral blood vessels due to the confined space of the skull and cerebrospinal fluid (CSF) [47]. As ICP increases, CSF pressure also rises, eventually surpassing MAP. Once ICP exceeds MAP, blood flow to the brain diminishes, leading to cerebral ischemia. This lack of oxygen supply is detected by the hypothalamus, which triggers a CNS ischemic response [34]. The hypothalamus activates the sympathetic nervous system, leading to increased CO, peripheral vasoconstriction, and a rise in BP. This compensatory mechanism restores blood flow to the brain by ensuring that MAP exceeds ICP, marking the first phase of the Cushing reflex [48, 49]. In the second phase, the elevated BP activates baroreceptors in the carotid sinus and aortic arch. These receptors send signals to the medulla, which triggers parasympathetic activation via the vagus nerve, resulting in a slowing of HR [45].

As proposed by Schmidt et al., CR is a physiological reflex in which fluctuations in ICP modulate sympathetic activity [5]. At modest or low ICP levels, it is a reversible determinant of efferent sympathetic outflow, playing as a physiological stressor. This perspective aligns with the observations of Guild et al., who proposed that CR, traditionally considered a protective reflex in the context of severe cerebral ischemia, may also be triggered by small but physiologically significant increases in the ICP [50]. Furthermore, emerging evidence supports a physiological ICP-mediated sympathetic modulation circuit and the existence of a possible intracranial baroreflex that regulates the sympathetic outflow based on CPP [51]. These findings have important implications, as they challenge the traditional perception of the Cushing response as merely a preterminal event and underscore the critical role of continuous ICP monitoring in the management of neurocritical care patients.

##### Trigemino-Cardiac Reflex (TCR):

TCR, also known as the oxygen-conserving reflex, is recognized as a brainstem-mediated physiological response triggered by stimulation of the sensory branches of the trigeminal nerve and characterized by an imbalance between sympathetic and parasympathetic activity [52]. It is observed in TBI as a result of direct craniofacial trauma [53]. Clinically, this reflex results in a triad of parasympathetic dysrhythmia (bradycardia), hypotension (systemic vasodilation and decreased cardiac output), and apnea (temporary disruption of the respiratory rhythm), often signaling elevated ICP or compression of brainstem structures [52, 54]. The mechanism of TCR involves a complex neural pathway. Sensory signals originating from the branches of the trigeminal nerve are transmitted through the Gasserian ganglion to the sensory nucleus of the trigeminal nerve in the brainstem. These signals are relayed via short interneuronal fibers within the reticular formation to the nucleus ambiguus and the dorsal motor nucleus of the vagus nerve. Recent research has explored the neuroprotective role of the TCR mechanism [53, 55]. This reflex induces rapid cerebrovascular vasodilation. By redistributing systemic blood flow to increase CBF, TCR acts as an endogenous neuroprotective response.

Two distinct pathways, the afferent-to-efferent neural communication, trigger TCR. The afferent pathway begins with sensory stimulation of the trigeminal nerve [11]. This stimulation, which can result from trauma or increased ICP, activates the sensory branches. These signals are transmitted to the spinal nucleus of the trigeminal nerve within the brainstem, where initial processing of the sensory input occurs. From here, the signals are relayed to two critical brainstem centers: the nucleus ambiguus and the dorsal motor nucleus of the vagus nerve. These nuclei serve as integration hubs for the reflex, processing the input and generating a coordinated parasympathetic response. The processed output is then directed through the vagus nerve, which mediates the reflex's physiological effects, such as bradycardia and hypotension [55, 56].

The efferent pathway is pivotal in producing its characteristic physiological effects. After sensory signals are processed in the brainstem, parasympathetic signals are transmitted via the vagus nerve to the cardiovascular system, leading to bradycardia and hypotension [11]**.** In bradycardia, the vagus nerve increases parasympathetic output to the heart, primarily targeting the SAN. This parasympathetic stimulation reduces the SAN's firing rate, leading to a slower heart rate. Additionally, vagal activity prolongs cardiac cycles by decreasing conduction velocity within the heart, further slowing the rhythm. The reduced HR also aligns with the reflex's neuroprotective role, as it decreases the heart's oxygen demand and conserves energy during stress or trauma.

In the context of TBI, TCR is particularly significant due to heightened sensitivity caused by brainstem ischemia, inflammation, or critically increased ICP. This heightened reflex can lead to substantial bradycardia, exacerbating the challenges of maintaining adequate CPP and potentially worsening brain injury through cerebral hypoperfusion. While the TCR’s evolutionary function may be to conserve oxygen during stress, its activation in TBI highlights severe autonomic dysregulation and is often a marker of poor neurological outcomes [56].

#### The Paradox of Autonomic Dysregulation in TBI

The brainstem is a critical component of the CNS, housing key autonomic centers, such as the nucleus tractus solitarius (NTS) and the vagal nuclei**,** which regulate HR, BP, and respiratory patterns [57]. In the context of TBI**,** structural or functional damage to the brainstem disrupts these autonomic centers, leading to significant cardiovascular dysregulation. One of the most profound manifestations of this is bradycardia, resulting from increased vagal activity due to brainstem compression or ischemia. This disruption impairs the heart's natural pacemaker mechanisms, reducing cardiac output and potentially leading to cerebral hypoperfusion [58]. Persistent bradycardia in TBI patients is often a marker of severe injury, highlighting the vulnerability of brainstem autonomic networks to trauma-induced damage [45].

In parallel, TBI often triggers sympathetic hyperactivity, driven by the brain's stress response to injury. This overactivation is characterized by a catecholamine surge, with excessive release of adrenaline and noradrenaline into the bloodstream [59, 60]. This response can cause transient tachycardia and elevated BP. While initially adaptive in maintaining perfusion, prolonged sympathetic overactivity can lead to detrimental effects, including cardiac dysfunction, endothelial damage, and exacerbation of ICP [3]. Chronic dysregulation of the autonomic system frequently follows, with oscillations between sympathetic dominance and parasympathetic overdrive**,** creating an unstable cardiovascular state that complicates patient management [3, 6].

This dual interplay of brainstem injury and sympathetic hyperactivity underscores the complexity of autonomic dysfunction in TBI [61, 62]. On the one hand, bradycardia reflects parasympathetic overactivation and brainstem damage, compromising perfusion and recovery. On the other hand, sympathetic hyperactivity adds a layer of cardiovascular instability that may worsen cerebral edema, increase metabolic demand, and exacerbate secondary brain injury [6, 63]. Together, these opposing forces highlight the need for a nuanced approach to monitoring and managing TBI patients.

### Mortality and Secondary Outcomes of Bradycardia in TBI

Literature specifically addressing the effect of HR on mortality in TBI patients is scarce. Although the predictive value of initial HR in trauma populations has been investigated in many studies, the significance of RB is still unknown. Often explained as a preterminal sign, RB has been linked to the failure of compensatory systems to sustain appropriate systemic perfusion.

TBI creates a unique situation in which bradycardia often accompanies hypertension as part of Cushing's reaction. Rising ICP in TBI causes a rise in systemic BP (to maintain CPP), accompanied by a compensatory reduction in HR. It remains uncertain what the slowed HR signifies for TBI patients, particularly those with high BP. Especially in hypertensive TBI patients, there has been very little clear evidence on whether RB is favorable or unfavorable.

A study by Mejaddam et al. reviewed hypertensive TBI patients and found that RB patients had higher mortality rates than tachycardia patients; however, this difference was not statistically significant in a small sample size study [64]. True bradycardia (TB) with HR < 60 bpm was shown to be the only HR condition that led to increased mortality in this study. The combination of hypertension with bradycardia suggests the presence of early Cushing's reflex. Moreover, the hospital stay of bradycardic patients was unexpectedly longer.

Prior studies have explored the prognostic significance and association between admission HR and clinical outcomes, suggesting that both bradycardia and tachycardia at presentation are associated with worse functional outcomes and higher mortality, independent of injury severity or other physiological parameters [33, 65]. Additionally, exposure to beta-blockers, which modulate sympathetic tone, has been associated with lower in-hospital mortality in patients with TBI. These studies primarily evaluated medication effects and did not specifically address dynamic changes in HR [66, 67]. A prior study identified reduced HR variability (HRV), defined as minimal fluctuations in HR (i.e., an indicator of physiological change), as a potential predictor of increased mortality following TBI [68, 69]. HRV is a specific measure of ANS functioning that quantifies beat-to-beat changes in HR. Notably, TBI of any severity is associated with lower HRV, which, in turn, predicts mortality [64].

During the acute phase of severe TBI, HR is modulated by multiple physiological and pathophysiological factors, including pain, heightened sympathetic activity, hypovolemia due to concurrent hemorrhagic injuries, and alterations in body temperature [70, 71]. Notably, a previous study evaluating the relationship between admission HR and mortality in moderate to severe TBI demonstrated a U-shaped association, with the lowest mortality observed in patients presenting with HR between 80 and 99 beats per minute [72].

Hypotension in the context of significant TBI is especially harmful as it may lower CPP and aggravate outcomes [72]. Moreover, the concurrent presence of bradycardia and hypertension-key components of Cushing’s sign- combined with an abnormal GCS motor score at presentation has been identified as a significant predictor of brain injury–neurosurgical intervention (BI-NSI) in school-aged children and adolescents following severe blunt trauma. BI-NSI is defined as either severe TBI necessitating urgent neurosurgical intervention or death attributable to presumed isolated severe TBI [19].

RB following blunt trauma in younger children has been proposed as an early clinical indicator of severe TBI [73]. Incorporating both SBP and HR has shown that a combination of elevated SBP and reduced HR was a significant predictor of brain injury with neurological signs of impairment (BI-NSI) among children aged 7–10 and 11–15 years, compared with age-matched peers with normal vital signs [19]. In adult blunt trauma patients, the presence of prehospital Cushing’s sign alongside altered mental status has been identified as a significant predictor of severe TBI, likely reflecting imminent herniation [19]. Early recognition of these signs may guide clinicians toward prompt neuroimaging and timely neurosurgical consultation, potentially improving outcomes by facilitating expedited interventions. Despite advances in diagnostic modalities, these traditional physiological markers remain valuable for the rapid identification of life-threatening intracranial pathology [19].

### Implications of RB: A Context-Dependent Phenomenon

The body typically responds to non-TBI trauma associated with hemorrhage with tachycardia as a compensatory physiological response to hypovolemia. A study conducted by Demetriades et al. revealed that RB occurred in 29% of hypotensive trauma patients who had SBP ≤ 90 mmHg along with HR ≤ 90 bpm and demonstrated lower crude mortality rates than tachycardic patients (21.7% versus 29.2%). [16]. Notably, RB appeared to be protective in subgroups with more severe injury profiles, including patients with an Injury Severity Score ≥ 16 or an Abbreviated Injury Scale score ≥ 3 for the chest or abdomen. They concluded that patients with severe injuries or major torso trauma who developed RB had better survival rates, indicating their bodies might maintain adequate stroke volume or enhanced vagal tone as an adaptive hemodynamic response [16].

Conversely, a large multicenter study by Ley et al. reported that RB served as a negative prognostic indicator [17]. The mortality rate was significantly higher among patients with bradycardia than among tachycardic patients (30.1% vs. 22.6%, *p* < 0.001), with an estimated adjusted odds ratio of 1.6. Patients with TB demonstrated the worst outcomes with a mortality rate of approximately 62%, while intermediate bradycardia (HR 60–90 bpm) resulted in a significantly lower risk of 9.7%. Notably, RB did not correlate with worse outcomes among patients aged 55 or older or those with better GCS scores (GCS ≥ 12) [17].

In another study, RB was not independently associated with increased mortality among hypertensive TBI patients [15]. In contrast, tachycardia was linked to an almost seven-fold higher mortality risk compared with patients without RB. This observation may represent an early manifestation of the Cushing reflex in the setting of impending brainstem herniation. Moreover, in univariate analysis, RB was associated with a higher risk of mortality, but this association didn't remain significant in multivariate analysis. TB turned out to be a strong independent predictor of death, similar to a study by Ley et al. [17]. Both RB and TB were associated with worse GOS scores. However, they were not associated with longer HLOS or ICU stays [15].

In contrast to general trauma patients, elucidating the physiological role of HR in patients with isolated TBI remains challenging. Among trauma patients, hypotension, commonly resulting from hemorrhage, triggers a compensatory tachycardic response to preserve cardiac output and maintain vital organ perfusion. The absence of this compensatory reflex implies an impaired homeostatic mechanism and is thus regarded as a poor prognostic indicator in non-TBI torso injuries.

In isolated TBI patients, the primary hemodynamic concern often shifts from systemic hypotension to compromised CPP. CPP may be jeopardized either by systemic hypotension, which reduces cerebral blood flow, or by severe hypertension, leading to elevated ICP through mechanisms such as vasogenic edema and intracranial hypertension [74]. The exact physiological implications of HR in this context are less clearly defined. HR alterations may reflect neural reflex pathways or autonomic dysregulation secondary to the brain injury itself. Understanding the interplay of these factors could enhance prognostication and inform individualized hemodynamic management strategies. Nevertheless, such studies are inherently limited by the restrictive indications for continuous ICP monitoring.

Overall, these conflicting results highlight the heterogeneity of RB and suggest that its prognostic significance is highly context dependent. This autonomic failure in progressive shock could be the cause of RB observed in hemorrhagic trauma patients. The decrease in HR may help maintain ventricular filling, but it may also indicate that circulatory collapse is imminent.

Therefore, RB should not be interpreted uniformly across different trauma-related conditions. A patient who presents with bradycardia and hypotension typically shows signs of hypovolemic shock along with altered vagal tone. In contrast, a patient with bradycardia and hypertension often presents with elevated ICP, leading to early herniation. These divergent mechanisms underscore the need for comprehensive clinical assessment and contextual interpretation. The different underlying mechanisms require healthcare providers to perform thorough clinical evaluations combined with appropriate context-based interpretation. Nevertheless, the prognostic role of RB in trauma remains controversial and warrants further prospective investigation.

#### Bradycardia in Traumatic SAH and SDH

 Bradycardia has also been reported in patients with subarachnoid hemorrhage (SAH), particularly in the context of TBI. Its occurrence often indicates Cushing's reflex, necessitating urgent intervention [75]. The pathophysiology of bradycardia in SAH is primarily attributed to central autonomic dysregulation, rather than intrinsic cardiac disease. It can occur independently or alongside other neuro-cardiac phenomena, such as QT prolongation, ST-segment changes, or stress-induced cardiomyopathy [4, 75, 76].

Recent studies have reported the prevalence of bradycardia in SAH to range between 20 and 30%. A cross-sectional study involving patients with spontaneous SAH reported bradycardia in 24.4% of cases, highlighting it as a common ECG abnormality [77]. In contrast, a review and meta-analysis reported a higher incidence (31.4%) of sinus bradycardia among patients with traumatic SAH, suggesting a greater autonomic impact in trauma-related cases [35, 78]. A study by Norberg et al. reported acute cardiac complications following SAH, including bradyarrhythmias, which significantly impacted long-term survival and increased the risk of subsequent cardiovascular events [79].

In TBI, bradycardia may serve as a subtle but clinically relevant marker of intracranial pathology, even in the absence of the full Cushing reflex. A case series by Faried et al. reported three patients who presented with acute traumatic SDH, and were initially managed conservatively but later developed isolated bradycardia in the absence of hypertension or respiratory changes as an early sign of hematoma progression, and neurological deterioration. Following timely neurosurgical decompression prompted by these autonomic changes, all three patients experienced complete recovery, suggesting that isolated bradycardia may serve as an early warning sign warranting urgent reassessment [80].

Moreover, a recent case report by Liu et al. reported a rare but clinically significant instance of paroxysmal bradycardia associated with severe headaches during the subacute phase of TBI [81]. A 34-year-old male with acute SDH, cerebral contusions, and SAH developed episodic bradycardia (30–40 bpm) on day 8 post-injury, each episode accompanied by a transient but intense headache. Notably, symptom resolution closely followed HR normalization. Although no direct injury to autonomic regulatory centers (e.g., insular cortex, brainstem) was evident, the authors postulated that evolving cerebral edema and neurohumoral dysregulation may have mediated the observed cardiovascular disturbance via the brain–heart axis rather than representing purely intracranial pathology [81].

Collectively, these findings indicate that bradycardia in TBI should not be overlooked as a secondary observation, whether it occurs early in the acute phase of injury as part of the rising ICP response or in the sub-acute phase as a sign of evolving secondary injury. Table 1 summarizes studies on bradycardia and TBI [13, 15, 18, 19, 35, 65, 77, 82–86]. Isolated bradycardia can serve as an important warning sign that carries significant prognostic and diagnostic value, which may help predict brain injury and guide timely treatment.

#### Bradycardia in Concomitant TBI and Spinal Injury

The incidence of simultaneous TBI and spine injury ranges from 4 to 8% [87]. Paiva et al. reported that, among 180 TBI patients, cervical spine injuries represented 86% of spine injury associated with TBI [88]. Shaikh et al. [84] showed that, among a total of 138 patients who were admitted to the ICU with high spinal cord injuries (HSCIs), 33% had prolonged bradycardia (a week or more). HSCI was defined as an injury affecting the spinal cord above the fourth dorsal vertebra (T3 or higher). At this level, it cuts the sympathetic outflow, and, at the same time, intact parasympathetic flow takes the upper hand, leading to parasympathetic dominance and various cardiac conduction abnormalities (i.e., bradycardia, heart block, and asystole). The higher the level of the spinal cord injury, the more profound the CVS involvement. In TBI with HSCI, 44.4% had prolonged bradycardia, vs. 34.4% had no prolonged bradycardia. Bradycardia during endotracheal suction occurred in both groups at rates of 62.4% and 2.2%, respectively. The authors did not comment on the contribution of bradycardia to TBI itself in this study, although 52 patients (38%) had concomitant TBI. Bradycardia due to concomitant TBI and HSCI has not been well addressed yet.

###  Future Directions

Despite growing recognition of brain–heart interactions in trauma, several critical gaps remain. Future research should prioritize the development of unified, context-specific definitions of relative bradycardia applicable to both general trauma and traumatic brain injury populations. Standardized reporting of confounding variables—including medication exposure, hypothermia, sedation, extracranial hemorrhage, and baseline cardiac disease—as well as precise timing of vital sign measurement, is essential to improve comparability across studies. Importantly, prospective, multicenter investigations are needed to determine whether RB provides independent prognostic or predictive value beyond established trauma severity **scores**, and whether its integration into early triage and neurocritical care algorithms can meaningfully improve clinical decision-making and outcomes. Despite growing recognition of BHIs in trauma, several critical gaps remain; therefore:

- Future research should prioritize the development of unified definitions of RB in trauma with and without TBI contexts, and in isolated TBI contexts.
- Standardized reporting of confounders and timing of vital sign capture, and prospective multicenter validation of whether RB patterns add predictive value beyond established trauma severity metrics.
- More robust studies on the utility of RB will be a call to include it within the advanced trauma life support (ATLS) guidelines and critical care to early risk stratification and proper triaging, aiming to timely pick up the imminent shock as well as impending traumatic brain herniation.

### Limitations

Despite these insights, current evidence remains limited by the heterogeneity of study designs, small sample sizes, and inconsistent definitions of bradycardia across studies, as shown in Table 1. Most of the data are heterogeneous and largely observational. The distinction between true bradycardia and relative bradycardia in trauma requires further prospective investigation to clarify their independent prognostic implications. It is worth addressing how to approach a patient with bradycardia following trauma, particularly TBI. Confounding factors such as sedatives, analgesics, negative chronotropic agents, hypothermia, extracranial hemorrhage, and baseline cardiac disease should be considered in the evaluation of bradycardia in addition to the differential diagnosis shown in Fig. 2.

## Conclusion

Bradycardia in TBI reflects a spectrum of underlying mechanisms with distinct clinical implications. RB and TB arise from different physiological pathways, including autonomic dysregulation, brainstem involvement, evolving intracranial pathology, or massive bleeding and should therefore not be interpreted uniformly across trauma contexts. Rather than representing a passive or pre-terminal phenomenon, bradycardia, particularly in TBI, may serve as an early marker of deteriorating intracranial dynamics or impending herniation. Recognition of bradycardia in the appropriate clinical context may facilitate earlier neuroimaging, prompt escalation of care, and timely neurosurgical intervention. Future studies incorporating standardized definitions, advanced neuromonitoring, and longitudinal outcome assessment are needed to clarify the prognostic role of bradycardia and refine its integration into trauma and neuro-critical care decision-making. A comprehensive understanding of bradycardia within the broader neuro-cardiac framework may contribute to more precise risk stratification and improved patient outcomes. Bradycardia, particularly RB, may be valuable if integrated in the ATLS guidelines as red-flag sign of serious underlying conditions.

## Data Availability

Not applicable as no data or analysis was generated.

## Declarations

### Clinical Trial Number

Not applicable, it is a review article.
